# Conserved Acidic Amino Acid Residues in a Second RNA Recognition Motif Regulate Assembly and Function of TDP-43

**DOI:** 10.1371/journal.pone.0052776

**Published:** 2012-12-26

**Authors:** Akemi Shodai, Akemi Ido, Noriko Fujiwara, Takashi Ayaki, Toshifumi Morimura, Miki Oono, Tsukasa Uchida, Ryosuke Takahashi, Hidefumi Ito, Makoto Urushitani

**Affiliations:** 1 Molecular Neuroscience Research Center, Shiga University of Medical Science, Shiga, Japan; 2 Department of Biochemistry, Hyogo College of Medicine, Hyogo, Japan; 3 Department of Neurology, Kyoto University, Kyoto, Japan; 4 Department of Neurology, Wakayama Medical University Graduate School of Medicine, Wakayama, Japan; International Centre for Genetic Engineering and Biotechnology, Italy

## Abstract

Accumulating evidence suggests that pathogenic TAR DNA-binding protein (TDP)-43 fragments contain a partial RNA-recognition motif domain 2 (RRM2) in amyotrophic lateral sclerosis (ALS)/frontotemporal lobar degeneration. However, the molecular basis for how this domain links to the conformation and function of TDP-43 is unclear. Previous crystal analyses have documented that the RRM2-DNA complex dimerizes under acidic and high salt conditions, mediated by the intermolecular hydrogen bonds of Glu246-Ile249 and Asp247-Asp247. The aims of this study were to investigate the roles of Glu246 and Asp247 in the molecular assembly of RRM2 under physiological conditions, and to evaluate their potential use as markers for TDP-43 misfolding due to the aberrantly exposed dimer interface. Unexpectedly, gel filtration analyses showed that, regardless of DNA interaction, the RRM2 domain remained as a stable monomer in phosphate-buffered saline. Studies using substitution mutants revealed that Glu246 and, especially, Asp247 played a crucial role in preserving the functional RRM2 monomers. Substitution to glycine at Glu246 or Asp247 induced the formation of fibrillar oligomers of RRM2 accompanied by the loss of DNA-binding affinity, which also affected the conformation and the RNA splicing function of full-length TDP-43. A novel monoclonal antibody against peptides containing Asp247 was found to react with TDP-43 inclusions of ALS patients and mislocalized cytosolic TDP-43 in cultured cells, but not with nuclear wild-type TDP-43. Our findings indicate that Glu246 and Asp247 play pivotal roles in the proper conformation and function of TDP-43. In particular, Asp247 should be studied as a molecular target with an aberrant conformation related to TDP-43 proteinopathy.

## Introduction

Recent advances in proteomics have allowed the identification of a new marker protein, TAR DNA-binding protein 43 kDa (TDP-43), for amyotrophic lateral sclerosis (ALS) and frontotemporal lobar degeneration (FTLD) [1], [2]. The features of TDP-43 pathology include the formation of cytosolic inclusions that are frequently ubiquitinated and phosphorylated, and the breakup of the carboxyl terminus into 25- or 35-kDa fragments [3]–[5]. These signs are observed exclusively in affected areas. The presence of genetic mutations in TDP-43 in a subpopulation of familial ALS patients further supports the primary role of TDP-43 in the pathogenesis of ALS [6]–[8].

TDP-43 is composed of an N-terminal nuclear localizing signal (NLS), two RNA-interacting domains (RRM1 and RRM2, with a nuclear export signal [NES] present in RRM2), and a C-terminus containing a glycine-rich domain. It is our consensus that the overexpression of carboxyl 25- and 35-kDa fragments containing the C-terminus is able to recapitulate many features of TDP-43 proteinopathy [9]–[12]. However, another study using the *C. elegans* model of TDP-43 proteinopathy found a definite motor phenotype only when the transgene contained both RRM1 and RRM2 [13]. More specifically, in several reports, the formation of cytosolic inclusions required the C-terminal portion of the RRM2 domain [11], [12], [14]. A different study using various truncation mutants of recombinant TDP-43 revealed that RRM2 conferred detergent-insolubility to an otherwise soluble N-terminal fragment containing RRM1 [15]. A mass spectrometric analysis of protease-resistant TDP-43 peptides showed that aggregate-core regions were concentrated in RRM2 and at the C-terminus [15]. Intriguingly, the caspase cleavage site for the 25 kDa fragments is also reportedly located in the RRM2 domain [10].

These lines of evidence imply that an aberrant conformation of the RRM2 domain might link to TDP-43 proteinopathy. Although many works have characterized RRM1 as the predominant domain for RNA processing [16], the exact role of RRM2 remains unknown. Kuo et al. performed intensive structural analyses of the RRM2-DNA complex, and found that murine RRM2-DNA cocrystalized under acidic and high salt conditions (2.0 M (NH_4_)_2_SO_4_, pH 4.2) through the hydrogen bonds of Glu246 (E246)-Ile249 (I249) and Asp247 (D247)-D247, which are located in the fourth β-strand [17]. Because the dimeric interface of RRM2 was observed only in the crystal structure of the RRM2-DNA complex, the authors suggested that the RRM2-DNA dimer may link to TDP-43 aggregate formation [17]. E246 and D247 have been proposed as an essential cleavage site to yield carboxyl fragments of TDP-43 [9]. In addition, residues 246–255 have been reported as a crucial aggregation core domain [18].

In light of the above studies and, in particular, the study by Kuo et al., we chose to investigate E246 and D247 of TDP-43 as a potential dimer interface and as markers for misfolded TDP-43, similar to the superoxide dismutase 1 (SOD1) epitope of dimer interface (SEDI) in SOD1 [19]. In the present study, we investigated the roles of E246 and D247 of TDP-43 under physiological conditions in the structure and function of the RRM2 domain and human TDP-43. Unexpectedly, we found that the soluble component of RRM2 was a stable monomer, regardless of DNA interaction, in which E246 and, especially, D247 played a role in monomer stability. Moreover, using a novel monoclonal antibody (mAb) against the RRM2 epitope containing D247, we found that D247 was exposed and served as a marker of cytosolic TDP-43 aggregates in cultured cells and ALS tissue.

## Materials and Methods

### Ethics Statement

The protocols for genetic analysis and neuropathological procedures were approved by and performed under the guidelines of our institutional ethics committee. Informed consent was obtained from all individuals or their guardians before the analysis.

### Plasmid construction and recombinant protein purification

Mammalian expression plasmids for human TDP-43 tagged with FLAG (pcDNA3-TDP-43-FLAG) or EGFP (pEGFP-N3-TDP-43) were constructed as described previously [20]. *E. coli* expression plasmids for the RRM2 domain (residues 190–265) of human TDP-43 were prepared with a commercially available kit (In fusion cloning kit, Clontech, Palo Alto, CA) and a conventional PCR protocol. In brief, PCR products obtained from pcDNA3-TDP-43-FLAG were subcloned into the BamHI site of pGEX6p-1 (GE Healthcare, Piscataway, NJ). Mutant RRM2 proteins at E246 and D247 were made with site-specific mutagenesis (QuickChange Lightning Site-Directed Mutagenesis kit, Agilent, La Jolla, CA). Deletion mutants of TDP-43-EGFP for RRM1 (ΔRRM1) or RRM2 (ΔRRM2) were generated by PCR, with primer pairs used to delete the nucleotides encoding these domains. All constructs were verified by DNA sequencing in a Big Dye Terminator 3.1 (Life Technologies, Carlsbad, CA). Primer sequences are provided in the Table S1.

For protein purification, the *E. coli* strain BL21(DE3)pLysS transformed with the expression plasmids was treated with 0.2 mM isopropyl thiogalactoside (IPTG) for 16 h at 22°C to induce protein expression. Proteins were cleaved from glutathione-S-transferase (GST) through digestion with a PreScission protease (GE Healthcare). The protein purity was validated by SDS-PAGE and Coomassie brilliant blue staining (Fig. S1). Proteins were dialyzed twice against phosphate-buffered saline (PBS) and stored in silicon-coated microtubes at −20°C.

### Thioflavin T assay

Thioflavin T (ThT) assay was performed to estimate β-sheet formation of RRM2 domain. Recombinant RRM2 proteins of ,WT or substitution mutants at E246/D247 were incubated for 10 min at 22 or 70°C. After 24 hr post-incubation at 4°C, the protein solution (0.1 mM in PBS) was reacted with an equimolar amount of 2-(4′-Methylaminophenyl)benzothiazole (BTA-1, Sigma), a derivative of ThT. Fluorescence was measured by the multi-plate reader (Tecan, Schweiz, Switzerland) at 440 nm for excitation and at 490 nm for emission.

### Determination of molecular size by size exclusion chromatography

Soluble RRM2 domains (2–3 mg/mL) with or without (TG)12 oligonucleotides, or extracts of cultured human embryonic kidney cells (HEK293A cells; Invitrogen, Carlsbad, CA) expressing TDP-43-FLAG in PBS were applied to a Superdex 75 10/300 column or to a Superose 12 10/300 column equilibrated with PBS, respectively (GE Healthcare). Samples were subjected to high-performance liquid chromatography (HPLC; AKTA Explorer 10S, GE Healthcare) at a flow rate of 0.5 mL/min in PBS. Before applying the column, protein samples were centrifuged for 20 min at 15000 g and 4°C to clear pellets. Recombinant proteins and (TG)12 oligonucleotides were detected by absorbance with 215 and 254 nm, respectively. TDP-43-FLAG was detected by Western blot with an anti-TDP-43 antibody. For the estimation of molecular weight, the columns were calibrated with thyroglobulin (669 kDa, GE Healthcare), ferritin (440 kDa, GE Healthcare), manganese superoxide dismutase (Mn-SOD; 88 kDa, NOF Corporation, Japan), bovine serum albumin (BSA; 66 kDa, Intergen, Milford, MA), ovalbumin (43 kDa, GE Healthcare), SOD1 (32 kDa, Sigma-Aldrich), myoglobin (17.6 kDa, Sigma-Aldrich), and aprotinin (6.5 kDa, Sigma) as protein standards.

### Cell culture, transfection, and immunofluorescence

HEK293A cells were cultured in Dulbecco's minimum essential media (DMEM) with 4.5 g/L glucose containing 10% fetal bovine serum (FBS) and penicillin/streptomycin. Human neuroblastoma SHSY-5Y cells [20] were cultured in DMEM/F12 Ham media containing 15% FBS, nonessential amino acids (NEAA), and penicillin/streptomycin. Cultures were kept at 37°C with 5% CO_2_ and 100% humidity. Expression plasmids for TDP-43 tagged with FLAG or EGFP at the carboxyl terminus were transiently transfected into cells with a FuGene HD transfection kit according to the manufacturer's instructions (Roche, Basel, Switzerland). Cells were analyzed at 48 h after transfection.

For immunofluorescence, cells were fixed with 4% paraformaldehyde (PFA) for 20 min at 22°C. After blocking in 5% normal goat serum in PBS with 0.2% TritonX100, cells were incubated with primary antibody. Cells were washed three times in PBS and reacted with a CF 568-conjugated secondary antibody at a dilution of 1∶1000 (Biotium, Hayward, CA). For counterstaining of nuclei, 4′,6-diamidino-2-phenylindole (DAPI, Nacalai Tesque, Kyoto, Japan) was used. Immunostained cells were observed with a confocal laser microscope (Nikon, Tokyo, Japan).

For the quantification of the number of cells with misfolded TDP-43, between 7 and 10 fluorescent photos were randomly taken and analyzed with Image J software (U. S. National Institutes of Health, Bethesda, MD). Cells with aggregates or nuclear exclusion were manually counted by an individual who was blinded to the identity of the transfected proteins.

### Polyacrylamide gel electrophoresis (PAGE) and Western blotting

Proteins were subjected to heat (70°C for 10 min) to investigate thermostability and susceptibility of oligomerization. For SDS-PAGE, protein samples were denatured in an SDS sampling buffer with 100 mM dithiothreitol (DTT) at 70°C for 20 min. Proteins were separated with a Tris-Bis polyacrylamide gel. After SDS-PAGE, gels were analyzed by Coomassie brilliant blue (CBB, Nacalai) staining or Western blot. For Western blotting, peroxidase-conjugated secondary antibodies (Jackson Immunoresearch, West Grove, PA) and an enhanced chemiluminescence kit (ECL; WestPico, Pierce, Rockford, IL) were used. To detect recombinant RRM2 proteins or full-length TDP-43 in cultured cells, rabbit polyclonal anti-TDP-43 antibody (ProteinTech Group Inc. Chicago, IL) was used (1∶1000). Mouse monoclonal anti-actin antibody (1∶1000) was purchased from Millipore (Billerica, MA). Rabbit polyclonal anti-glyceraldehyde-3-phosphate dehydrogenase (GAPDH) antibody (1∶1000) was purchased from Santa Cruz (Santa Cruz, CA).

To examine the effect of amino acid substitution on the detergent solubility of TDP-43 proteins, transfected cells were homogenized in lysis buffer (50 mM Tris-HCl pH 7.4, 150 mM NaCl, 10% glycerol, 1% TritonX100) and centrifuged for 60 min at 20,400 g at 4°C. Supernatants were mixed with SDS sampling buffer (adjusted to 2% SDS and 100 mM DTT). After a single rinse with the lysis buffer, pellets were resuspended in 2% SDS sampling buffer with 100 mM DTT with five cycles of sonication for 20 seconds each. The supernatant and pellet were heat-denatured at 70°C for 20 min and analyzed by Western blotting as the detergent-soluble and -insoluble fractions, respectively.

To quantify insolubility, the membranes were incubated with rat mAb anti-GFP (Nacalai Tesque, Kyoto) for TDP-43-EGFP and reblotted with an anti-actin antibody and analyzed with imaging software (Image J). TDP-43-EGFP proteins were standardized by actin, in which the ratio of TDP-43-EGFP to actin was obtained in either the detergent-soluble (ratio-sol) or detergent-insoluble (ratio-insol) fraction. Moreover, each ratio was standardized by the averaged ratio of the WT. The insolubility index was obtained by dividing ratio-insol by ratio-sol, and was expressed as fold change compared to WT.

### Exon 9 skipping assay

The RNA splicing of TDP-43 was estimated by using an exon 9 skipping assay with a TG13T5 minigene reporter plasmid, as reported previously but with minor modifications [16], [21]. HEK293A cells were transiently cotransfected with TDP-43-EGFP (0.5 µg per well) and pTG12 (3 µg per well, a generous gift from Drs. Emanuele Buratti and Francisco E. Baralle) in 6-well dishes by using FuGene HD (Roche). The mRNA was harvested by using Trizol and a commercially available RNA purification kit (Purelink RNA purification kit; Invitrogen). Five micrograms of total RNA and random hexanucleotide primers were used for reverse transcription (Superscript III, Invitrogen). As a negative control, reverse transcriptase-free samples were tested. Exon 9 skipping was estimated from the densitometric analysis of the spliced to unspliced fragments observed with a PCR reaction using the following primer pairs: 5′ TAGGATCCGG TCACCAGGAA GTTGGTTAAA TCA 3′ and 5′ CAACTTCAAG CTCCTAAGCC ACTGC 3′. The efficiency of exon 9 skipping was analyzed by densitometry with Image J.

### Generation of mAb

Antigen peptides were designed within QSLCGEDLIIKGISVHISNA (241–260AA) and injected into C57Bl/6 mice after emulsification in Complete Freund's adjuvant (CFA). Hybridomas were generated by fusing mouse spleens and P3-X63-Ag8-U mouse myeloma cells through a conventional protocol with 50% polyethylene glycol. The first screening was positive/negative selection against recombinant wild type (WT) and then E246G/D247G RRM2 proteins by ELISA. The second selection involved immunohistochemistry with spinal cord sections of sporadic ALS patients who were previously confirmed to have TDP-43 pathology.

### Immunoprecipitation

To clarify the affinity of the generated mAb against TDP-43 in cells, HEK293A cells transfected with TDP-43-FLAG were analyzed by immunoprecipitation and Western blotting. Protein G-coated magnetic beads (20 µL) (Dynal, Invitrogen) were coupled to 3B12A by incubation with 1 mL of the hybridoma supernatant at 4°C for 16 h. Total cell lysates from transfected HEK293A cells in RIPA buffer without SDS were incubated with 3B12A coupled to protein G magnetic beads in the same buffer at 4°C overnight. Beads were washed five times in RIPA containing 0.1% SDS and then eluted with 2× SDS sampling buffer containing 100 mM DTT at 70°C for 20 min. Immunoprecipitates were analyzed by Western blot with a rabbit polyclonal anti-FLAG antibody (Abnova).

### Immunohistochemistry of ALS spinal cords

Postmortem lumbar spinal cords from five patients with neuropathologically proven sporadic ALS (mean age: 64.6 years) and three age-matched neurologically normal subjects (mean age: 68.7 years) were analyzed. The mean postmortem delays of the ALS and control groups were 9.0 and 9.4 h, respectively. During autopsy, spinal cords were removed, and blocks of the lumbar levels of the spinal cords were immediately placed in 10% buffered formalin and embedded in paraffin. No pathological changes were noted in the spinal cords from control subjects.

For immunohistochemistry, after heat retrieval by autoclaving (10 min at 121°C in 10 mM sodium citrate buffer), 6 µm thick sections were incubated overnight at 4°C with either 3B12A diluted 1∶250 with PBS containing 3% BSA (PBS-BSA) or rabbit polyclonal antibody against amino acids 1–260 of TDP-43 (ProteinTech) diluted 1∶2000 with PBS-BSA. Bound primary antibodies were detected with a Vectastain Elite ABC kit (Vector Laboratories, Burlingame, CA) with 3,3′-diaminobenzidine tetrahydrochloride used as the chromogen. The staining specificity was confirmed by replacing the primary antibody with the appropriate amount of PBS-BSA. No reaction product deposits were seen in these control-stained sections.

For the double immunofluorescent study, the spinal cord sections were incubated simultaneously with rabbit polyclonal anti-ubiquitin antibody (Dako, Glostrup, Denmark) and 3B12A mAb. Sections were washed three times in PBS, incubated with a mixture of anti-mouse IgG-Alexa fluor 488 and anti-rabbit IgG Alexa fluor 568 (Invitrogen), and observed by fluorescence microscopy (Olympus System Microscope BX53, Olympus, Tokyo, Japan).

### Statistics

Comparison of multiple experiments was performed by one-way analysis of variance (ANOVA) with Newman-Keuls test with the Prism statistical software package (GraphPad, La Jolla, CA). A *p*-value<0.05 was judged as significant.

## Results

### E246 and/or D247 residues are crucial to preserve the monomeric state of recombinant RRM2 protein

**Figure 1:**
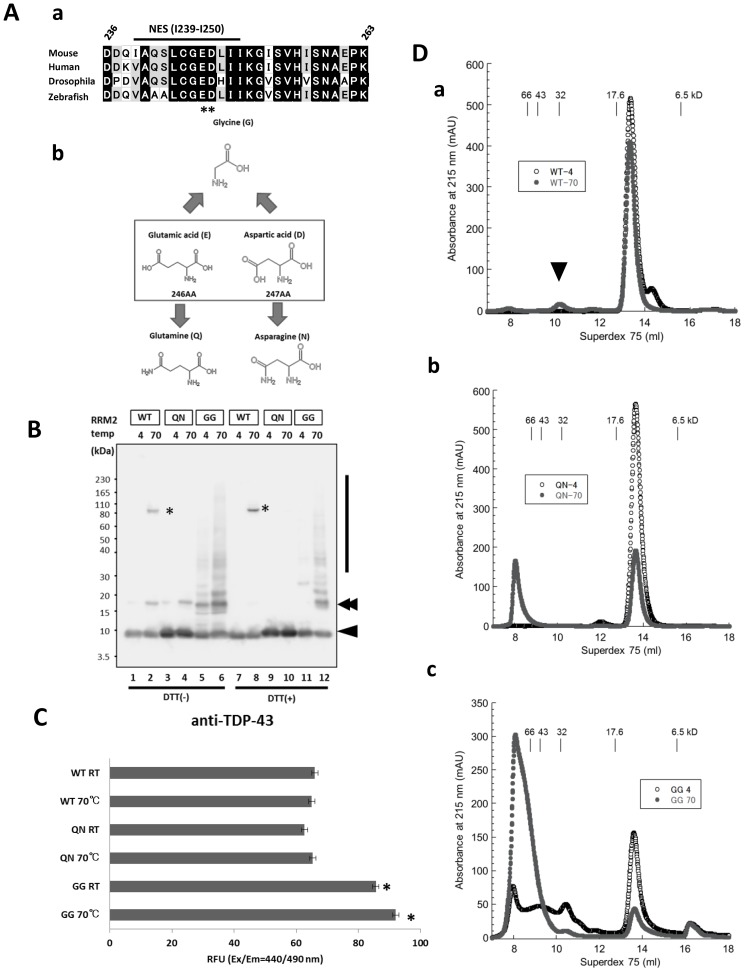
Pivotal role of Glu246 and Asp247 in the conformation of the RRM2 domain of TDP-43. **A. a,** Scheme showing the alignment of RRM2 subdomains (residues 232–270 in human TDP-43) in multiple species. Glu246 (E246) and Asp247 (D247) are preserved across all species. **b,** Chemical properties of amino acid substitution mutants of E246 or D247. E246Q and D247N were used to create substitution mutants with minimal alteration of side chains. E246G and D247G were designed so that the effects of the side chains were eliminated, and the flexibility of the amide bonds was increased. **B.** Western blot analysis of recombinant RRM2 proteins of wild-type (WT), E246Q/D247N (QN), and E246G/D247G (GG), using an anti-TDP-43 rabbit polyclonal antibody that recognizes the RRM2 domain (Proteintech). The E246G/D247G mutant RRM2 showed marked oligomerization even when incubated at 4°C (vertical line). Note that considerable RRM2 dimers or oligomers were dissociated into monomers in the presence of DTT. Arrowhead and double arrowheads indicate RRM2 monomer and dimers, respectively. Asterisk possibly indicates heat-related high molecular complexes comprising RRM2 domain. **C.** Thioflavin T (ThT) fluorescence assay showing amyloid fibril formation of RRM2 with mutations in E246 and D247. Each value indicates averaged RFU of ThT with standard error of mean from triplicates. *p<0.01 vs. RRM2 WT at RT by one-way ANOVA with Newman-Keuls test. **D.** Superdex 75 size exclusion chromatography of recombinant RRM2 domain of WT (**a**), E246Q/D247N (QN, **b**), and E246G/D247G (GG, **c**). **a**, WT RRM2 alone was exclusively monomeric in its native condition (unfilled circle). Heat denaturation at 70°C for 10 min induced higher molecular assembly (filled circle). Arrowhead indicates the RRM2 oligomer. **b**, E246Q/D247N (QN) mutant RRM2 without stress was predominantly monomeric (unfilled circle). Heat denaturation markedly increased the ratio of oligomers to monomers (filled circle). **c,** E246G/D247G (GG) mutant existed as a mixture of monomer and oligomers at the baseline condition (unfilled circle). Higher molecular species were prominently induced by heat denaturation (filled circle). Molecular size markers are as follows: bovine serum albumin (66 kDa), ovalbumin (43 kDa), copper zinc superoxide dismutase (32 kDa), myoglobin (17.6 kDa), and aprotinin (6.5 kDa).

Previous work by Kuo et al. [17] documented that the murine RRM2-DNA complex is dimerized under acidic (pH 4.2) and high salt (2.0 M (NH_4_)_2_SO_4_) conditions, through the hydrogen bonds of E246-I249 and D247-D247. To investigate whether this dimerization links to pathological conditions in TDP-43 proteinopathy, we generated recombinant proteins for the RRM2 domain (aa 190–265) of human WT TDP-43 and several substitution mutants of E246 and/or D247, which are conserved acidic amino acids among various species (Fig. 1A, a). We investigated the molecular weight of these RRM2 proteins in the physiological buffer condition (PBS, pH 7.2). E246 and D247 were substituted to amino acids with similar side chains, E246Q/D247N (QN), to minimize any potential structural influence. We also created a double glycine residue mutant, E246G/D247G (GG), to generate a deformed version of RRM2 (Fig. 1A, b).

Western blot analysis under denaturing conditions showed that recombinant RRM2 proteins of the WT and QN mutant migrated exclusively to around 10 kDa, a calculated monomeric size, under the physiological (Fig. 1B, 4°C, lanes 1, 3, 7, 9) and heat-stress conditions to investigate thermostability and susceptibility to oligomerization (Fig. 1B, 70°C for 10 min, lanes 2, 4, 8, 10). On the other hand, the GG mutant formed SDS-resistant high molecular weight (HMW) intermediates or oligomers (Fig. 1B, lanes 5, 6, 11, 12, vertical line) in addition to monomers. Of note, RRM2 dimer was monomerized in the presence of dithiothreitol (DTT) (Fig. 1B, lanes 2, 4, 5, 8, 10, 11), indicating the partial involvement of disulfide-bond in RRM2 dimerization by oxidation. Moreover, a Thioflavin T (ThT) assay revealed that GG mutant acquired higher ThT interaction than other forms (Fig. 1C). These findings indicated that E246 and D247 were located at the fragile region, the substitution of which could be amyloidogenic. [22].

Size exclusion chromatography on a Superdex 75 column demonstrated that nucleotide-free RRM2 (WT) migrated in a single peak of ∼10 kDa (Fig. 1D, a), which indicated that RRM2 existed as a monomer in physiological solution. Exposure to 70°C for 10 min decreased the monomeric peak to 90% and induced additional small peaks that were consistent with the dimer size (arrowhead). The RRM2 (QN) mutant was also a soluble monomer when incubated at 4°C (Fig. 1D, b, unfilled circles). However, exposure to 70°C for 10 min generated a soluble oligomer that was eluted in the void fractions with size >100 kDa (Fig. 1D, b, filled circles). The RRM2 (GG) mutant without any stress at 4°C was collected in several fractions consisting of monomers and variously sized oligomers (Fig. 1D, c, unfilled circles). Heat-stress of the RRM2 (GG) mutant promoted the formation of soluble HMW complexes (Fig. 1D, c, filled circles).

We also examined whether RRM2 forms molecular complexes by perfluoro-octanoic acid (PFO)-PAGE, which preserves the protein-protein interactions of both cytosolic and membranous proteins [23]. In contrast to the Western blotting of the denatured samples, in which high molecular species of RRM2 proteins were detected only in the GG mutant, but not in QN mutant, PFO-PAGE showed that the QN mutant also displayed high molecular shifts as well as GG mutant (Fig. S2). These results strongly suggested that the side chain structures of E246 and D247 played important roles to preserve the conformation of the soluble RRM2 domain under physiological conditions, and that damage to these residues may have induced the oligomerization of RRM2.

### D247 is the dominant residue for regulating the conformation of RRM2

**Figure 2:**
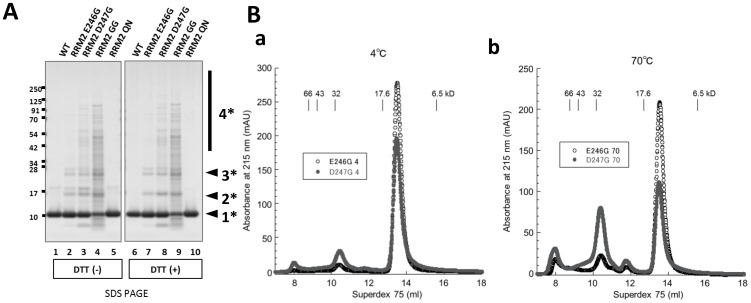
D247 predominantly governs the conformation of RRM2. **A.** Coomassie staining of recombinant RRM2 protein with either single or double mutation(s) at E246 and D247 incubated for 24 hr at 4°C by denaturing SDS-PAGE. The effects of oligomerization were greater with the single mutant at D247 than at E246. Oligomerization is most prominent in the double E246G/D247G mutant. **B.** Size exclusion chromatography of RRM2 proteins of E246G and D247G incubated at 4°C (a) and 70°C for 10 min with 24 hr post incubation at 4°C. (b). The effect of oligomerization is more pronounced in the D247G mutant RRM2 than in the E246G in both the 4°C and 70°C conditions. Molecular size markers are as follows: bovine serum albumin (66 kDa), ovalbumin (43 kDa), superoxide dismutase 1 (32 kDa), myoglobin (17.6 kDa), and aprotinin (6.5 kDa).

We next compared the effects of amino acid replacement of either E246 or D247 in the oligomerization of RRM2 by PAGE and Superdex 75 size exclusion chromatography. To examine which of these two amino acid residues contributes to the proper conformation of RRM2, a single substitution mutant of each residue to glycine was generated. SDS-PAGE revealed that D247G displayed SDS-resistant and DTT-independent oligomers with higher molecular weight than the E246G mutant RRM2 (Fig. 2A). Superdex 75 size exclusion chromatography of soluble RRM2 proteins also indicated that the D247G mutant (Fig. 2B, b) was more susceptible to oligomerization than the E246G mutant (Fig. 2B, a) at both 4°C and 70°C.

### Substitution of E246/D247 induces the formation of aggregates and oligomers of full-length TDP-43

**Figure 3:**
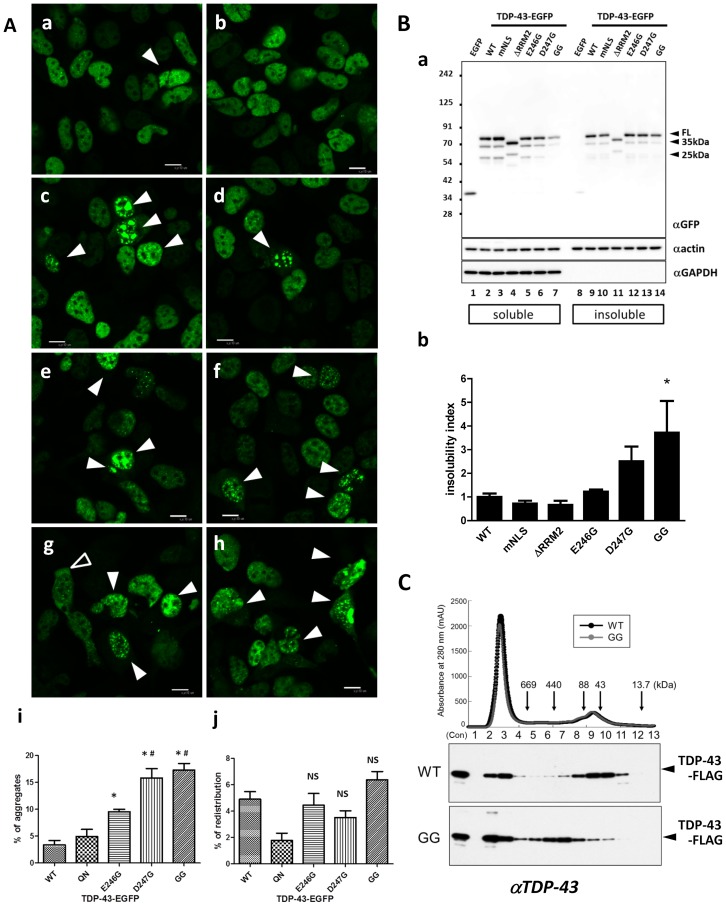
Substitution mutants of full-length TDP-43 at E246 and D247 are readily misfolded. **A.** Confocal micrographs of HEK293A cells overexpressing EGFP-fused full-length TDP-43 (**a, b,** wild-type (WT), **c, d,** E246G, **e, f,** D247G, **g, h,** E246G/D247G). **i, j,** Percentages of transfected HEK293A cells harboring multiple puncta or inclusions (**i**, arrowheads) or displaying nucleus-excluded TDP-43 (unfilled arrowhead). (**j**). Scale bar indicates 30 µm. Data were expressed as the mean ± SEM (N = 7–10). *p<0.05 vs. WT, #*p*<0.05 vs. E246G by one-way ANOVA with Newman-Keuls test. NS indicates not significant vs. WT. **B. a,** Western blotting showing the increased detergent-insolubility of TDP-43 with mutations at E246/D247, with defective nucleus localizing signal (mNLS), or devoid of RRM2 domain (ΔRRM2). Lysates from HEK293A cells transiently transfected with TDP-43-EGFP were separated into 1% TritonX100-soluble or -insoluble fractions. Top panel, anti-GFP; middle panel, anti-actin; bottom panel, anti-GAPDH. The GAPDH blot validates the successful separation between detergent-soluble and -insoluble components. **b,** Quantified insolubility of TDP-43-EGFP proteins with or without mutation at E246/D247 to glycine. Relative TDP-43-EGFP in the detergent-soluble or -insoluble fraction was obtained from the ratio of the GFP density to actin density from the densitometric value in each fraction (designated as insoluble TDP or soluble TDP, respectively). Insolubility index was obtained from the ratio of insoluble TDP to soluble TDP, and each value was standardized by the average ratio of WT. Data were expressed as the mean ± SEM of four experiments.*p<0.05 vs. WT TDP-43-EGFP by one-way ANOVA with Newman-Keuls test. **C.** Size exclusion chromatography and Western blotting indicating the existence of oligomeric and monomeric states of full-length TDP-43 in cells. WT and E246G/D247G (GG) TDP-43-FLAG genes were expressed in HEK293A cells. Cells were sonicated in PBS, and the supernatants were fractionated by a Superose 12 column (10/300) at a flow rate of 0.5 mL/min in PBS. Fractionated cell extracts were applied to Western blotting by anti-TDP-43 (Proteintech). Mutant TDP-43 (GG) proteins were collected in a larger fraction than 88–440 kDa. The molecular size markers thyroglobulin (669 kDa), ferritin (440 kDa), Mn-SOD (88 kDa), ovalbumin (43 kDa), and RNase (13.7 kDa) were eluted under the same conditions. Abs280 is presented to show the equal amount of proteins between WT and the GG mutant in each fraction.

We further investigated the effect of the substitution of E246/D247 on the conformation and functions of full-length (FL) TDP-43. HEK293A cells were transiently transfected with WT TDP-43-EGFP or various mutants with substitutions at E246 and/or E247 (E246G, D247G, E246G/D247G (GG), or E246Q/D247N (QN)). According to confocal fluorescence microscopy analysis, substitutions of E246 and/or D247 promoted the formation of multiple punctate aggregates or large inclusions, mostly in the nucleus but with occasional cytosolic distribution (Fig. 3A, a–h; arrowheads indicate aggregates). Quantification by cell counting revealed that substitution mutants at E246 and/or D247 showed more cells with TDP-43 aggregates, and that mutants with D247G showed a marked propensity for aggregate formation (Fig. 3A, i). Although E246 and D247 are located in NES [24], there was no clear trend as to cytosolic redistribution (Fig. 3A, j), indicating that these residues do not play crucial role to regulate NES. Human neuronal SHSY-5Y cells displayed similar but less prominent aggregate formation than HEK293A cells, probably because of their lower protein expression levels (Fig. S3).

We next examined the effect of E246/D247 substitution of TDP-43 on the detergent (1% TritonX100) solubility of TDP-43. EGFP-tagged TDP-43 of WT, mutants with defective NLS (mNLS), and several substitution mutants of E246/D247 were overexpressed in HEK293A cells. Using an anti-GFP antibody, we performed Western blot analyses of the denatured cell lysates, which had been separated into detergent-soluble and -insoluble fractions. The results revealed that all types of TDP-43 were predominantly monomers. The expression levels of TDP-43-EGFP containing RRM2 (GG) were obviously lower than those of the others. Comparable amounts of the detergent-insoluble species of TDP-43-EGFP (GG) were obtained relative to the other types (Fig. 3B, a). This finding indicated that this mutant was prone to aggregation. The insolubility index was determined as the ratio of detergent-insoluble to detergent-soluble TDP-43-EGFP/actin. TDP-43-EGFP containing RRM2 (GG) was significantly insoluble compared to WT (Fig. 3B, b), and D247G showed a clear trend of higher insolubility than that of WT.

Effects of the substitution of the E246 and D247 residues on the molecular assembly of TDP-43 under physiological conditions were investigated by size fractionation. At 48 h after transfection of TDP-43-FLAG containing WT or GG RRM2, transfected HEK293A cells were resuspended in PBS, briefly sonicated, and centrifuged at 17,400× g for 5 min at 4°C. The supernatants were introduced into a Superose 12 column for size exclusion. As shown in Fig. 3C, both TDP-43 proteins were eluted as large protein complexes in the void fractions, with size >2 MDa, as reported previously [25]. Although WT TDP-43 was eluted with monomer and dimer sizes, GG TDP-43 was eluted with an apparent molecular mass of ∼440 kDa, which indicated that GG TDP-43 had a tendency to form oligomers or protein complexes. Of note, Western blot analysis of the full-length TDP-43-EGPP with E246G and/or D247G in the denatured conditions also did not display obvious oligomers regardless of DTT as observed in recombinant RRM2 proteins (data not shown). It is possible that RRM2 assembly was affected by other domains in full-length TDP-43. Moreover, together with the result from gel-filtration analysis, it is indicated that high molecular TDP-43 species was mediated by protein-protein interaction.

### Aberrant oligomerization affects the nucleotide interaction and RNA splicing efficiency of TDP-43

The role of E246/D247 in the RNA splicing of TDP-43 was investigated with the established exon 9 splicing cassette of cystic fibrosis transmembrane conductance regulator (CFTR) [16]. HEK293A cells were doubly transfected with pTG12 and pEGFP-TDP-43 (WT, defective NLS (mNLS), deletion mutants of RRM1 or RRM2, E246G/D247G (GG), or E246Q/D247N (QN)). Then, cDNA was obtained by using random primers from the total RNA in the cells and analyzed by PCR to detect the spliced and unspliced fragments.

**Figure 4:**
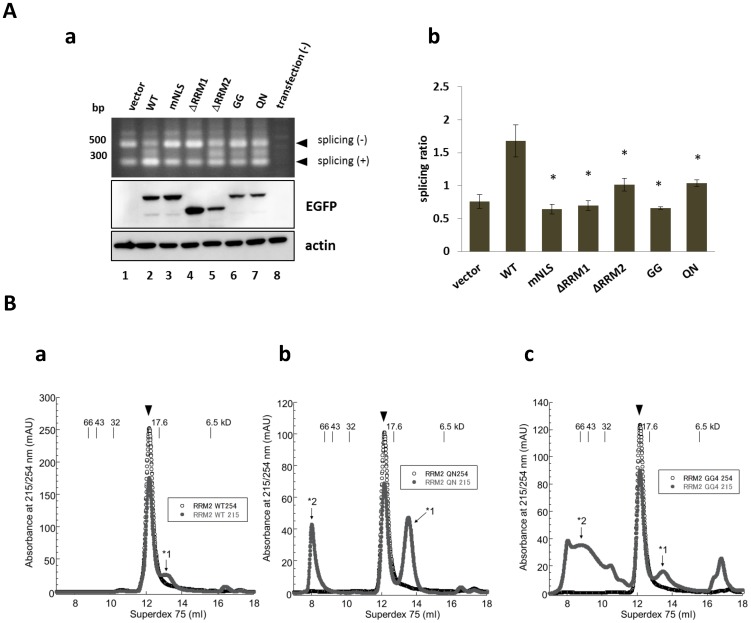
Oligomerization affects the nucleotide interaction and RNA splicing efficiency of TDP-43. **A.** Exon 9 skipping assay showing that mutations at E246 and D247 affect the RNA splicing activity of full-length TDP-43. **a**, Agarose gel electrophoresis of PCR products (top). Western blot analysis of the total cell lysates using anti-EGFP (middle) and -actin (bottom) antibodies was also shown. **b**, Quantification of spliced and unspliced fragments using densitometry. Each value is the ratio of spliced to unspliced PCR products. Data is mean ± standard error of mean from triplicates. *p<0.01 vs. Wild-type (WT) TDP-43 by one-way ANOVA with Newman-Keuls test. **B.** Size exclusion chromatography for recombinant RRM2 proteins of WT (a), or mutants with E246Q/D247N (QN, b) or E246G/D247G (GG, c), and for (TG)12 oligonucleotides. Mixtures of RRM2 mutants and (TG)12 oligonucleotides were centrifuged at 15,000× g for 20 min and subjected to a Superdex75 (10/300) column at a flow rate of 0.5 mL/min in PBS. Only the RRM2 monomer showed a molecular shift with the (TG)12 oligonucleotides to a single peak, indicating their association (arrowheads). Molecular size markers are as follows: bovine serum albumin (66 kDa), ovalbumin (43 kDa), superoxide dismutase 1 (32 kDa), myoglobin (17.6 kDa), and aprotinin (6.5 kDa). *1 indicates free monomeric RRM2, *2 indicates oligomeric RRM2. Note that there is no peak for free (TG)12, indicating all the (TG)_12_ was bound to RRM2 monomers.

WT TDP-43 efficiently promoted the splicing of exon 9 compared to the vector control (Fig. 4A, a, b, lanes 1 and 2). The RRM1 deletion mutant (ΔRRM1) completely reversed this splicing effect (lane 4), whereas ΔRRM2 partially reversed it (lane 5). Interestingly, the cytosolic form of TDP-43 with a defective nuclear localizing signal (mNLS) also showed a defect in splicing, consistent with a previous report showing that both RRM1 and the NLS are required for this splicing [9]. The RRM2 (GG) mutant displayed a splicing defect as extensive as the ΔRRM1 mutant. On the other hand, the RNA splicing of the RRM2 (QN) mutant was higher than that of RRM2 (GG), but comparable to that of ΔRRM2, despite that the protein expression levels of QN and GG were comparable in the Western blotting (Fig. 4A,a, middle and bottom panels). This finding indicated that the structure of the side chain at these residues crucially affected the RNA splicing function of RRM2.

To obtain insight into the molecular basis of how E246/D247 regulates the RNA splicing of TDP-43, the interaction efficiency of various RRM2 mutants with thymidine-guanine 12-repeats ((TG)_12_), which indirectly reflects the splicing efficiency of RRM domains, was examined [16]. Recombinant WT-RRM2 was mixed with oligonucleotides with (TG)_12_ for 1 h at 37°C before loading onto a Superdex 75 column. This pretreatment generated a single peak detected at 215 and 254 nm, the optimal wavelengths for proteins and nucleotides, respectively. The molecular mass was 20 kDa, corresponding to the combined size of monomeric RRM2 and (TG)_12_ (Fig. 4B, a, arrowhead) and indicating their interaction. When WT-RRM2 and (TG)_12_ were separately applied to the size chromatography column, they were collected in different fractions (data not shown). We further investigated the influence of the substitution of E246/D247 on the DNA-binding affinity of RRM2. Monomeric RRM2 (QN) maintained its binding affinity with (TG)_12_ even when exposed to 70°C for 10 min, as shown by the merged signals for absorbance at 215 and 254 nm (Fig. 4B, b, arrowhead). However, oligomeric RRM2 (QN) remained with no absorbance at 254 nm (Fig. 4B, b, *2), which indicated that RRM2 (QN) oligomerization obliterated the DNA-binding affinity. The same result was obtained in the case in the RRM2 (GG) mutant with heat denaturation. As shown in Fig. 4B, c, monomeric RRM2 (GG) interacted with (TG)_12_, although oligomeric RRM2 (GG) could not bind (TG)_12_. A small amount of residual free WT, QN and GG monomers still remained as a separate peak (Fig. 4B, a, b, c, *1), which is most probably because the amount of RRM2 monomer overwhelmed that of (TG)_12_. These results clearly demonstrated that the monomeric conformation of RRM2 was crucial to preserve the DNA-binding affinity.

Collectively, our results strongly indicated that the side chain structure of E246 and D247 played pivotal roles in preserving the conformation and RNA metabolism activity of TDP-43, although E246 and D247 are not essential for interacting with DNA.

### A monoclonal Ab against E246/D247 in RRM2 recognizes cytosolic TDP-43 with a defective NLS

Our results revealed that substitution mutants at E246 and D247 led to an aberrant conformation and defective DNA/RNA processing of the RRM2 domain and TDP-43. A previous crystal study demonstrated that, under acidic and high salt conditions, the RRM2-DNA complex is dimerized through two hydrogen bonds of E246-I249 and D247-D247. Based on these findings, we hypothesized that the regional environment around these residues would be affected by protein misfolding of TDP-43 in ALS, as has been observed in the case of dimer interface residues of SOD1 in familial ALS [19].

We therefore generated a mAb against 21 peptides containing E246 and D247 (Q241-A260, Fig. S4A), which contained a partial α-helix and two β-strands (β4 and β5) [17]. Hybridoma cells secreting IgG that reacted with WT RRM2 but not with RRM2 (GG) were selected (Fig. S4A). From this screening, we obtained one mAb clone, which we designated as 3B12A. This clone highly reacted with recombinant RRM2 protein and FL TDP-43 proteins purified from *E. coli* (Fig. S4B) but did not react with RRM1 or other control proteins (BSA and SOD1, Fig. S4C). Interestingly, the 3B12A reactivity to RRM2 was completely eliminated when D247 of RRM2 was substituted to glycine, identifying D247 as a crucial epitope for the binding (Fig. S4D). 3B12A strictly recognized the side chain structure of D247 because the E246Q/D247N substitution of RRM2, which mimicked WT RRM2 without physical stress (e.g., heat denaturation), completely abolished the binding affinity of 3B12A (Fig. S4E). 3B12A was unavailable for Western analysis, due to its conformation-specific recognition of TDP-43.

**Figure 5:**
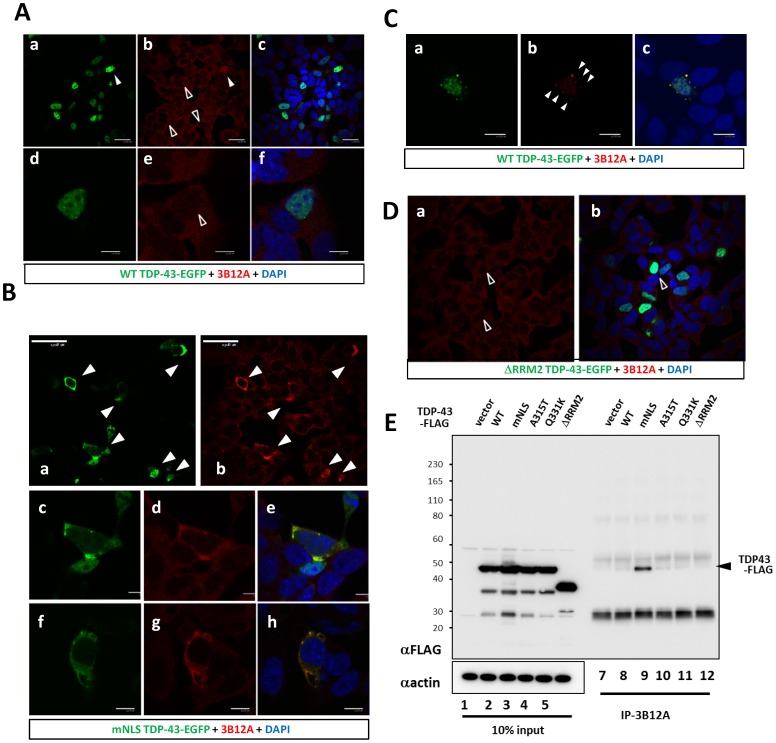
3B12A recognizes cytosol-redistributed TDP-43. **A–D**, SHSY-5Y cells were transiently transfected with TDP-43-EGFP of wild type (WT), mutants with defective NLS (mNLS), or deletion mutant of RRM2 deletion (ΔRRM2) (EGFP shown as green). At 48 h after transfection, cells were fixed and stained with 3B12A (red). DAPI was used for counterstaining (blue). **A. a–f**, Transfected or endogenous WT TDP-43 was rarely stained by 3B12A (unfilled arrowheads). Occasionally, cells with very high fluorescence were labeled (arrowhead). **B.** Cytosolic redistributed TDP-43 (mNLS) was preferentially stained by 3B12A (arrowheads). 3B12A recognized the mNLS mutant of TDP-43-EGFP even at moderate expression levels, regardless of aggregate formation. **c–e** are high power fields of **a–b**. **C.** Nuclear-excluded WT TDP-43 is recognized by 3B12A. WT TDP-43-EGFP expressing SHSY-5Y cells exposed to 5 µM lactacystin were fixed and stained with 3B12A (arrowheads). **D.** No reactivity of 3B12A to TDP-43-EGFP devoid of RRM2 (ΔRRM2) (unfilled arrowheads). **E.** Immunoprecipitation experiment showing that 3B12A preferentially recognized NLS-defective TDP-43 in cell lysates. HEK293A cells were transiently transfected with WT, mNLS, or FALS mutant (A315T and Q331K) forms of TDP-43-FLAG. Total lysates were immunoprecipitated with the 3B12A. Western blot analysis using a rabbit polyclonal anti-FLAG antibody showed that 3B12A predominantly recognized the defective NLS, but more weakly recognized the WT and FALS-linked mutant forms of TDP-43.

We performed immunofluorescent analysis using various types of human TDP-43-transfected cells to investigate whether the 3B12A mAb was able to detect the epitope including the D247 residue. 3B12A did not stain most of the exogenous WT or endogenous nuclear TDP-43 proteins (Fig. 5A, unfilled arrowheads). Surprisingly, however, 3B12A recognized most, but not all, of the cytosolic TDP-43 with a defective NLS (mNLS) of either type of homogenous distribution or aggregates (Fig. 5B, filled arrowheads). When nuclear WT TDP-43 had a high fluorescence intensity, it was stained by 3B12A (Fig. 5A, filled arrowheads), which implied that a high concentration of WT TDP-43 affected its conformation or 3B12A modified the WT TDP-43 structure. These results indicated that cytosolic TDP-43 had a different conformation from the nuclear one, and that the epitope including D247 was accessible to 3B12A in the cytosolic TDP-43.

Aberrant cytosolic WT TDP-43 aggregates under exposure to proteasome inhibitor lactacystin were also stained by 3B12A (Fig. 5C). We validated these findings by changing the secondary antibody for 3B12A from CF 568 to 488 and using CF 568 for TDP-43-FLAG, which is overexpressed in HEK293A cells (Fig. S5). Nonspecific interactions to the high fluorescence were excluded because 3B12A did not react to ΔRRM2 TDP-43 (Fig. 5D). Familial ALS-linked mutant TDP-43 proteins tended to be more stained than WT, but were not so clearly labeled by 3B12A as cytosolic TDP-43 (Fig. S6).

We further examined the different reactivity of 3B12A between nuclear and cytosolic TDP-43 by immunoprecipitation experiments using the cell lysates of transfected cells. Lysates from HEK293A cells overexpressing FLAG-tagged TDP-43 of WT or mNLS were immunoprecipitated with 3B12A and probed with anti-FLAG antibody. 3B12A efficiently pulled down mNLS TDP-43, whereas the immunoprecipitation of nuclear TDP-43 of WT or FALS-linked mutants was relatively weak in comparison. 3B12A did not interact with the TDP-43 mutant lacking RRM2 (Fig. 5E). There is a possibility that 3B12A recognizes DNA/RNA-free TDP-43, since cytosolic species were preferentially stained. We thus examined the immunoreactivity of 3B12A against TDP-43 in the presence or absence of (TG)_12_ by sandwich ELISA. As shown in Fig. S7, 3B12A recognized TDP-43 proteins regardless of DNA interaction.

### Pathological hallmarks of ALS are immunoreactive to 3B12A mAb

**Figure 6:**
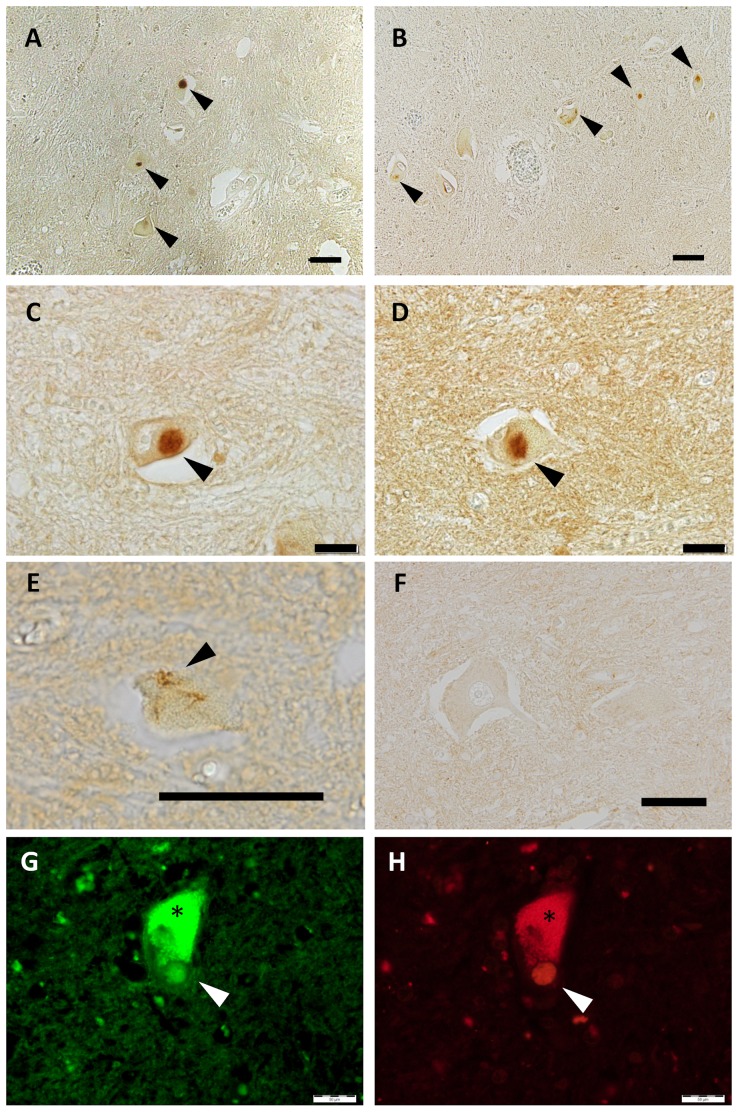
3B12A recognizes TDP-43 inclusions from the spinal cords of ALS patients. **A–F.** Immunohistochemistry of anterior horn cells using 3B12A from four sporadic ALS patients (**A–E**) and representative negative control from three subjects. Arrows indicate pathologic structures of ALS, such as Lewy body-like hyaline inclusions (**A–D**) and skein-like inclusions (**E**). Nuclei from non-ALS control subjects are not labeled by 3B12A (**F**). Asterisk indicates lipofuscin. Panels B and D are from the same patients. **G, H.** Representative double immunofluorescence imaging of round cytosolic inclusions in the close section to panel A. Spinal cord sections were stained by 3B12A (green) and anti-ubiquitin antibody (red). Scale bar indicates 50 µm.

We performed immunohistochemical analyses of ALS tissues using 3B12A. 3B12A stained various cytosolic inclusions, such as Lewy body-like hyaline inclusions (Fig. 6A–D) and skein-like inclusions (Fig. 6E). Immunostaining of serial sections using rabbit polyclonal anti-TDP-43 antibody confirmed that the 3B12A-positive inclusions were also TDP-43-positive (Fig. S8). 3B12A did not stain normal nuclei in samples from control subjects (Fig. 6F). A double immunofluorescence study revealed that several of the 3B12A-positive inclusions were positive for ubiquitin (Fig. 6G, H). These results suggested that cytosolic redistribution altered the conformation of TDP-43, which exposed D247 to be recognized by 3B12A. In other words, D247 may be a potential immunogenic marker of ALS-relevant TDP-43 inclusion. However, not all inclusions were stained by 3B12A, which implied that D247 accessibility was altered in the process of aggregate formation in some cases. Approximately, 80% of TDP-43-positive inclusions were recognized by 3B12A.

## Discussion

A previous crystal study documented that E246 and D247, located in the NES of the RRM2 domain of TDP-43, mediate dimer formation of the RRM2-DNA complex via hydrogen bonds. In this report, we have focused on these two conserved acidic amino acids as candidate regulators of the molecular assembly. We hypothesized that these residues may serve as misfolding markers for TDP-43, similar to the dimer interface of mutant SOD1 proteins in familial ALS [19]. To test our hypothesis, we investigated the conformation of the functional unit of RRM2 under physiological conditions, because the previous crystal analysis by Kuo et al. was performed in an acidic and high salt solution [17]. The results of this analysis may provide clues as to how the native TDP-43 deviates from the conformational equilibrium, leading to pathological inclusions. Unexpectedly, our gel filtration analysis revealed that recombinant WT RRM2 existed as a monomer in physiological solution, regardless of DNA interaction (Fig. 4B, a) or heat treatment (Fig. 1D, a). Plausible reasons to explain this apparent discrepancy may include differences in the species of TDP-43 that were analyzed or the tags used for recombinant proteins (no-tagged human here and His-tagged murine in the previous report). Unexpectedly, the substitution of E246/D247 to Q246/N247 (QN) in RRM2 induced the oligomerization of RRM2 after heat stress. Moreover, glycine substitution at E246/D247 (GG) led to oligomerization even without any stress (Fig. 1D).

We also showed that, to interact with the nucleotide for splicing, it is pivotal for RRM2 to be in the monomeric state. In fact, monomers preserved the interaction with DNA regardless of mutations, whereas oligomers induced by the E246/D247 substitution abolished the (TG)_12_-binding affinity (Fig. 4B). Furthermore, the D247G mutant was more susceptible to oligomerization than E246G (Fig. 2). These results indicated that E246 and especially D247 were critical for the proper structure and functions of TDP-43, and that the side chain structure governed the conformation of TDP-43. Such aggregates were basically mediated by protein-protein interactions. However, the RRM2 oligomers were not easily dissociated into monomers in reducing SDS-PAGE, which indicated that β sheet-mediated oligomerization [22] was involved in the misfolding process of the substitution mutant RRM2 at E246/D247. The E246G/D247G mutant of TDP-43 readily formed soluble oligomers and acquired hydrophobicity in cultured cells (Fig. 3C), consistent with the results of RRM2 domain analysis. Moreover, the exon 9 skipping assay revealed that the E246/D247 mutation decreased the splicing effect (Fig. 4A). It was suggested that the decreased splicing effect might have been caused by decreased RNA-binding activity, based on the aberrant conformation of RRM2 with the disruption in E246/D247 (Fig. 4B).

Intriguingly, our novel mAb, 3B12A, which recognized D247 as an epitope, strongly reacted with recombinant RRM2 or full-length TDP-43 proteins by ELISA, but poorly reacted with nuclear WT TDP-43 in cells or unaffected human spinal cord sections. On the other hand, cytosolic TDP-43 in cultured cells or pathological hallmarks of ALS specimens, such as Lewy body-like hyaline inclusions or skein-like inclusions, were stained by 3B12A (Fig. 6). These results suggested that the epitope of 3B12A was not exposed when WT TDP-43 existed in the nucleus, and that the cytosolic redistribution of WT TDP-43 may have caused exposure of the epitope, including D247. Consistent with this notion, a previous report documented that E246/D247 is one of the cleavage sites to yield TDP-43 fragments in ALS/FTLD [9]. Moreover, the results from our mutation study with single amino acid substitutions clearly indicated that D247 had a greater impact on preventing the aberrant oligomerization of RRM2 than E246 did.

The RRM domains of TDP-43 have five β-strands, whereas those in many other RNA-binding proteins have four, which may underlie its propensity towards amyloid formation [17]. E246 and D247 are normally located on the fourth β-strand in the RRM2 domain, which is adjacent to the fifth β-strand. Therefore, we think that E246 and D247 are “hidden” or covered by protein complexes in the native conformation of TDP-43. However, if these residues are exposed to the outside under noxious conditions, then the fibril formation of TDP-43 might occur. Very interestingly, 3B12A seemed to recognize early pathogenic species of TDP-43 but not normal nuclear TDP-43 because the cytosolic TDP-43 detected by 3B12A was generally not ubiquitinated or phosphorylated (Fig. S9). A possible interpretation for this finding is that the 3B12A mAb detects the early pathogenic conformation of TDP-43. However, not all of the cytosolic aggregates were immunoreactive to 3B12A in ALS sections or cultured cells. It is possible that the side chain of D247 is covered in the process of aggregate formation.

Despite the consensus that cytosolic TDP-43 inclusions are among the pathological hallmarks of ALS, knowledge on the role of the regional structures in the conformation of TDP-43 is limited. An increasing body of evidence indicates that TDP-43 proteinopathy underlies motor neuron degeneration caused by diverse genetic defects in various hereditary ALS, including ubiquilin 2 [26], profilin-1 [27], optineurin [28], valosin-containing protein (VCP) [29], and C9ORF72 [30]. Moreover, mutations in TDP-43 cause ALS [6]–[8], indicating that an abnormal conformation of TDP-43 initiates pathogenic cascades toward motor neuron death. TDP-43 is actively shuttled between the nucleus and cytosol in the physiological state [31], [32]. The identification of specific amino acids that serve as a marker of pathogenic TDP-43 will contribute to our understanding of TDP-43 proteinopathy. In this sense, 3B12A might contribute to the early detection of pathogenic conformers of ALS/FTLD-relevant TDP-43.

Our studies using substitution mutants of E246/D247 clearly showed that the side chain structure of these residues strictly governed the functional conformation of the RRM2 domain. The exact role of E246/D247 in the pathogenesis of ALS has yet to be determined. Clarification of the chemical modifications or binding proteins focusing on E246/D247 will advance our understanding of TDP-43 proteinopathy. Given the advancement of various promising therapeutic studies, the early diagnosis of ALS/FTLD is inevitable. Therefore, our finding that E246 and D247 may serve as molecular markers for early pathogenic cytosolic TDP-43 species could contribute to the future improvement of therapeutic interventions. In light of the recent notion that various pathogenic proteins found in neurodegenerative diseases are propagated similar to prion proteins, molecular targeting using an antibody such as 3B12A might be a promising approach to capture the intercellular species. Further investigation is necessary to clarify the initial events that induce the misfolding of TDP-43 and to expand our understanding of and cure TDP-43 proteinopathies, such as ALS and FTLD.

## Supporting Information

Figure S1 **SDS-PAGE analysis of de novo recombinant RRM2 proteins of WT, various substitution mutants at E246 and/or D247 purified from E coli.** 5 µg of recombinant RRM1 proteins of wild-type (WT), E246Q/D247N (QN), and E246G/D247G (GG) under denaturing conditions with 100 mM DTT. Gels were stained with Coomassie brilliant blue.
(PDF)

Figure S2 **PFO-PAGE study to investigate the molecular size of the RRM2 domain in WT or various mutations of E246/D247.** After heat treatment (at 37 or 70°C) or overnight agitation, recombinant RRM2 proteins of wild-type (WT), E246Q/D247N (QN), and E246G/D247G (GG) under denaturing conditions, were incubated in 1% perfluoro-octanoic acid (PFO) sampling buffer for 1 h and then separated by a 15% PAGE in buffer containing 0.5% PFO. Gels were stained with Coomassie brilliant blue (CBB). RRM2 with substitutions at E246 and D247 was readily oligomerized, especially by heat stress at 70°C for 10 min.
(PDF)

Figure S3 **Effect of deletion of RRM2 domain or substitution at E246 and D247 of TDP-43 on the cellular distribution and morphology of SHSY-5Y cells.** Confocal micrographs showing the expression patterns of various full-length TDP-43 constructs with or without mutations (QN, E246Q/D247N; GG, E246G/D247G) or TDP-43 devoid of the RRM2 domain (ΔRRM2) in human neuronal SHSY-5Y cells. GG and D247G mutants show more nuclear and, occasionally, cytosolic aggregates. Scale bar indicates 10 µm.
(PDF)

Figure S4 **Generation of mouse monoclonal antibody against D247.** A, Schematic representation of antigen design and screening procedure for mAb production. Supernatant of hybridoma obtained from mice, which had been immunized with peptides containing E246 and D247 (241–260 aa), was tested by ELISA for reactivity against recombinant WT RRM2 and E246G/D247G RRM2. B, ELISA showing the reactivity of 3B12A mAb to recombinant proteins for full-length TDP-43 and WT RRM2 domain. C–E, ELISA showing that 3B12A recognizes WT RRM2, with a specific reactivity for D247. 3B12A recognized recombinant RRM2 but did not recognize RRM1, mouse superoxide dismutase 1 (SOD1), or bovine serum albumin (BSA) (C). 3B12A recognized WT RRM2 and E246G mutant RRM2 but did not recognize D247G or E246G/D247G mutants (D). 3B12A did not recognize RRM2 containing the E246Q/D247N mutations, which substituted amino acids with similar side chains (E).
(PDF)

Figure S5 **3B12A mAb effectively stains cytosolic TDP-43.** Confocal immunofluorescent micrographs of HEK293A cells expressing TDP-43-FLAG of wild-type (A, D) and defective NLS (mNLS, H). In D–F, HEK293A cells were treated with 20 mM lactacystin to induce nuclear exclusion of WT TDP-43. Cells were doubly stained with antibodies against FLAG (red) and 3B12A (green). Scale bar indicates 10 µm.
(PDF)

Figure S6 **Familial ALS-linked TDP-43 is marginally recognized by 3B12A.** Confocal immunofluorescent micrographs of SHSY-5Y cells expressing mutant TDP-43-EGFP (green, A, A315T; B, Q331K) which were stained by 3B12A mAb(red). Scale bar indicates 30 µm.
(PDF)

Figure S7 **3B12A mAb interacts with TDP-43 regardless of DNA interaction.** A sandwich ELISA showing that 3B12A recognizes recombinant TDP-43 protein regardless of its interaction with biotin-labeled (TG)12 repeat oligonucleotides. Rabbit polyclonal anti-TDP-43 antibody was coated onto ELISA plate to capture recombinant TDP-43 protein. 3B12A was used for detection antibody after 1 hr incubation with biotin-labeled (TG)12 oligonucleotide (blue). The (TG)12, which interacted with captured TDP-43 was also quantified by obtaining peroxidase activity of streptavidin-conjugated horseradish peroxidase without 3B12A application (green).
(PDF)

Figure S8 **3B12A stains TDP-43–positive round cytosolic inclusions.** Serial sections from a different ALS patient from those in Fig. 6 were stained with 3B12A mAb (A) and anti-TDP-43 antibody (B). The round inclusion in the cytosol of motor neurons is immunoreactive to anti-TDP-43 and 3B12A antibodies. Scale bar indicates 50 µm.
(PDF)

Figure S9 **Non-aggregated forms of cytosolic TDP-43 are not ubiquitinated or phosphorylated.** Confocal micrographs used for immunofluorescence analysis of SHSY-5Y cells that were transiently transfected with TDP-43-EGFP or the defective nuclear localizing signal form of TDP-43-EGFP (mNLS). After fixation with 4% paraformaldehyde, cells were stained with anti-ubiquitin (A–C) or anti-phosphorylated TDP-43 at S409/S410 (D–F) antibody. Note that the mNLS mutant form of TDP-43 is not labeled by these antibodies. Scale bar indicates 10 µm.
(PDF)

Table S1 Primer pairs used for plasmid construction.
(PDF)

File S1 Supplementary Materials and Methods.
(PDF)
