# Supplementary figures and images for: Conserved Acidic Amino Acid Residues in a Second RNA Recognition Motif Regulate Assembly and Function of TDP-43
Source: PLoS One. 2012 Dec 26;7(12):e52776. doi: 10.1371/journal.pone.0052776 (PMC3530536; doi:10.1371/journal.pone.0052776)

Figure S1

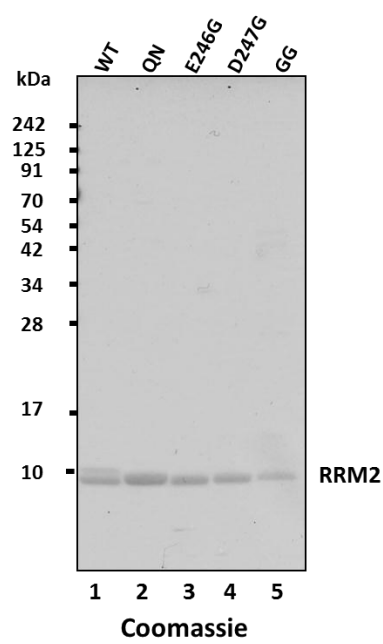

Supplement: Figure S1 — SDS-PAGE analysis of de novo recombinant RRM2 proteins of WT, various substitution mutants at E246 and/or D247 purified from E coli. 5 µg of recombinant RRM1 proteins of wild-type (WT), E246Q/D247N (QN), and E246G/D247G (GG) under denaturing conditions with 100 mM DTT. Gels were stained with Coomassie brilliant blue. (PDF) [file pone.0052776.s001.pdf]

Figure S2

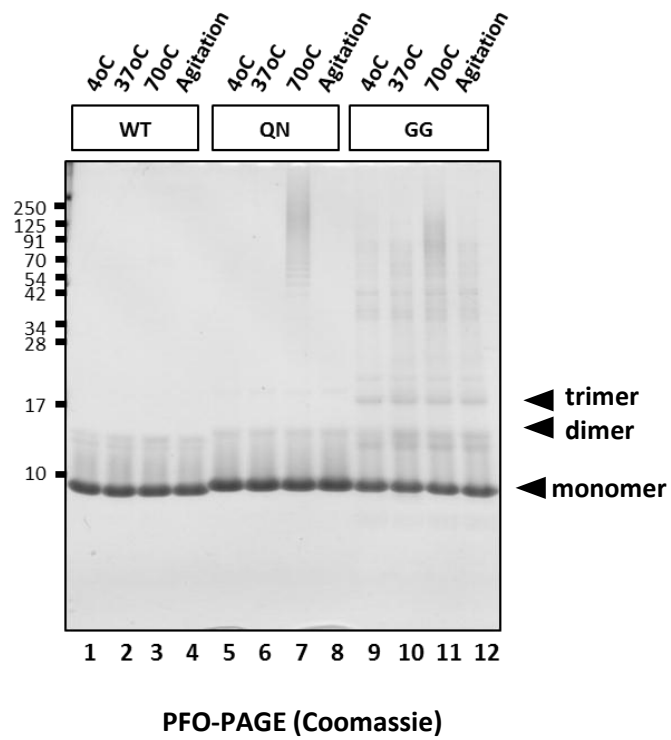

Supplement: Figure S2 — PFO-PAGE study to investigate the molecular size of the RRM2 domain in WT or various mutations of E246/D247. After heat treatment (at 37 or 70°C) or overnight agitation, recombinant RRM2 proteins of wild-type (WT), E246Q/D247N (QN), and E246G/D247G (GG) under denaturing conditions, were incubated in 1% perfluoro-octanoic acid (PFO) sampling buffer for 1 h and then separated by a 15% PAGE in buffer containing 0.5% PFO. Gels were stained with Coomassie brilliant blue (CBB). RRM2 with substitutions at E246 and D247 was readily oligomerized, especially by heat stress at 70°C for 10 min. (PDF) [file pone.0052776.s002.pdf]

**Figure S3**

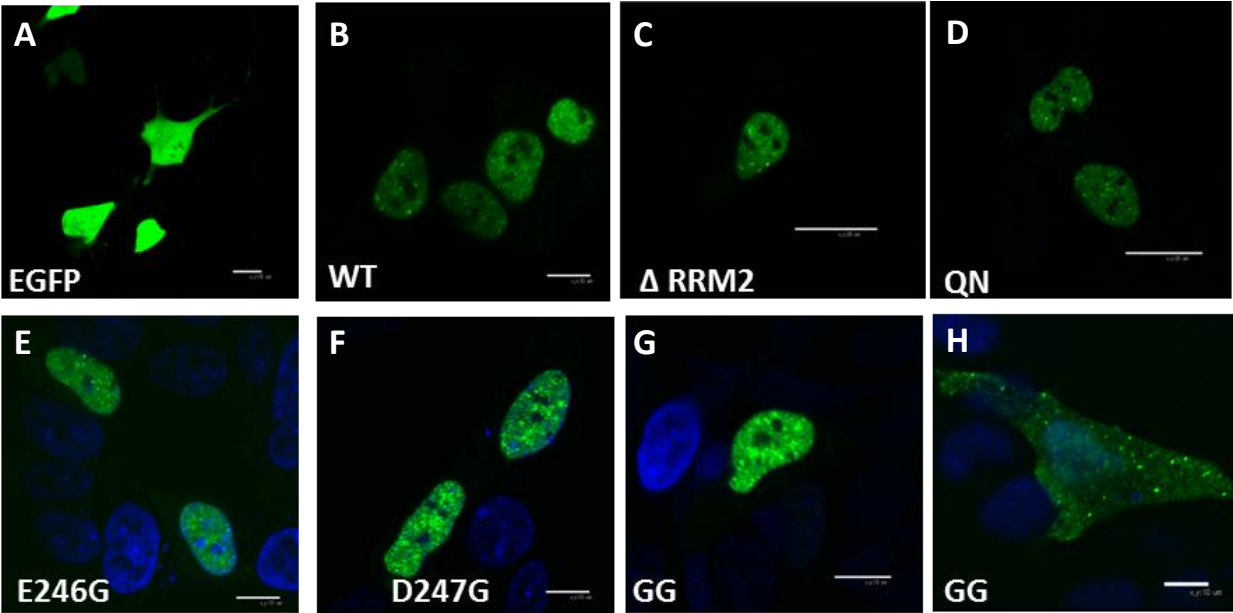

Supplement: Figure S3 — Effect of deletion of RRM2 domain or substitution at E246 and D247 of TDP-43 on the cellular distribution and morphology of SHSY-5Y cells. Confocal micrographs showing the expression patterns of various full-length TDP-43 constructs with or without mutations (QN, E246Q/D247N; GG, E246G/D247G) or TDP-43 devoid of the RRM2 domain (ΔRRM2) in human neuronal SHSY-5Y cells. GG and D247G mutants show more nuclear and, occasionally, cytosolic aggregates. Scale bar indicates 10 µm. (PDF) [file pone.0052776.s003.pdf]

Figure S4

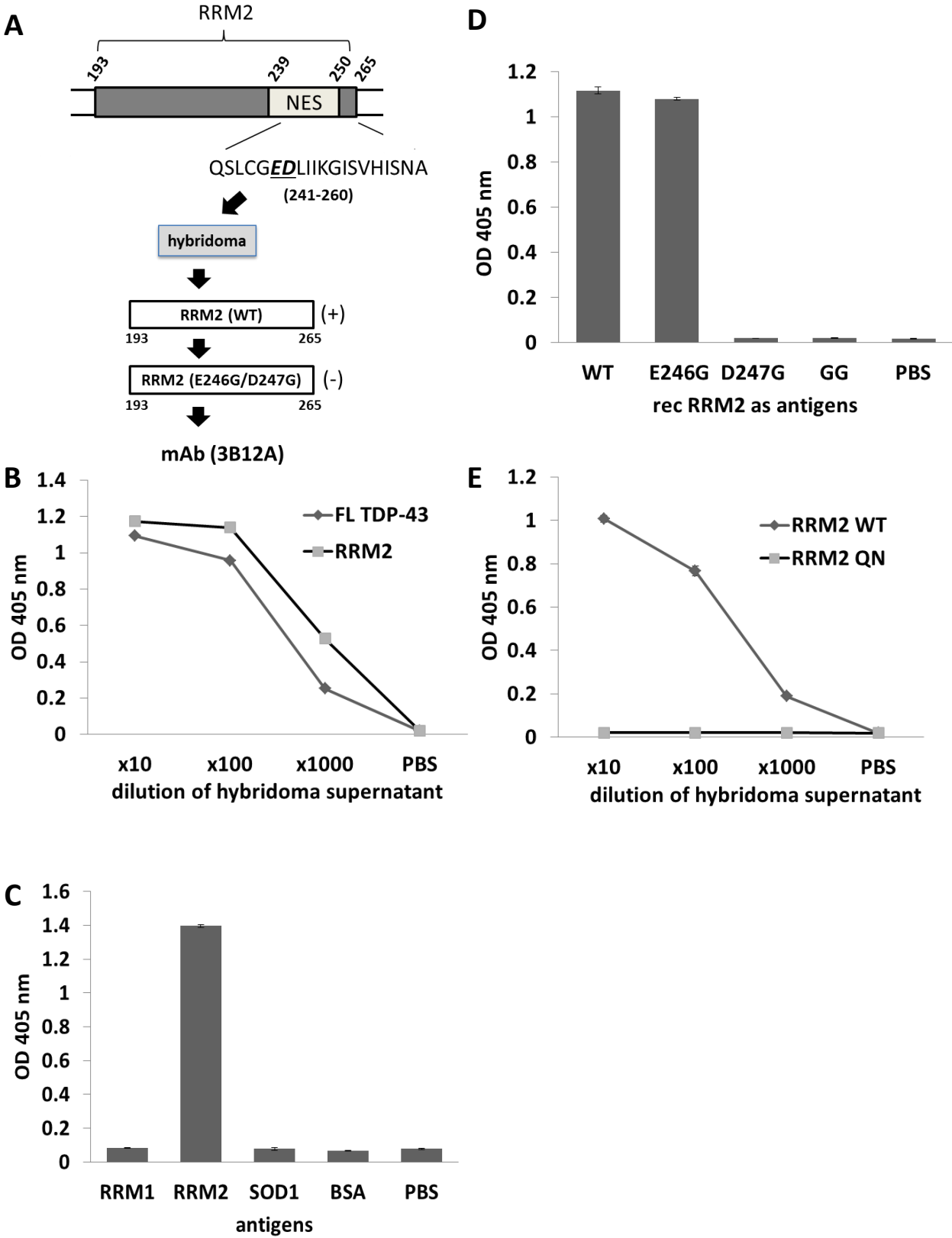

Supplement: Figure S4 — Generation of mouse monoclonal antibody against D247. A, Schematic representation of antigen design and screening procedure for mAb production. Supernatant of hybridoma obtained from mice, which had been immunized with peptides containing E246 and D247 (241–260 aa), was tested by ELISA for reactivity against recombinant WT RRM2 and E246G/D247G RRM2. B, ELISA showing the reactivity of 3B12A mAb to recombinant proteins for full-length TDP-43 and WT RRM2 domain. C–E, ELISA showing that 3B12A recognizes WT RRM2, with a specific reactivity for D247. 3B12A recognized recombinant RRM2 but did not recognize RRM1, mouse superoxide dismutase 1 (SOD1), or bovine serum albumin (BSA) (C). 3B12A recognized WT RRM2 and E246G mutant RRM2 but did not recognize D247G or E246G/D247G mutants (D). 3B12A did not recognize RRM2 containing the E246Q/D247N mutations, which substituted amino acids with similar side chains (E). (PDF) [file pone.0052776.s004.pdf]

Figure S5

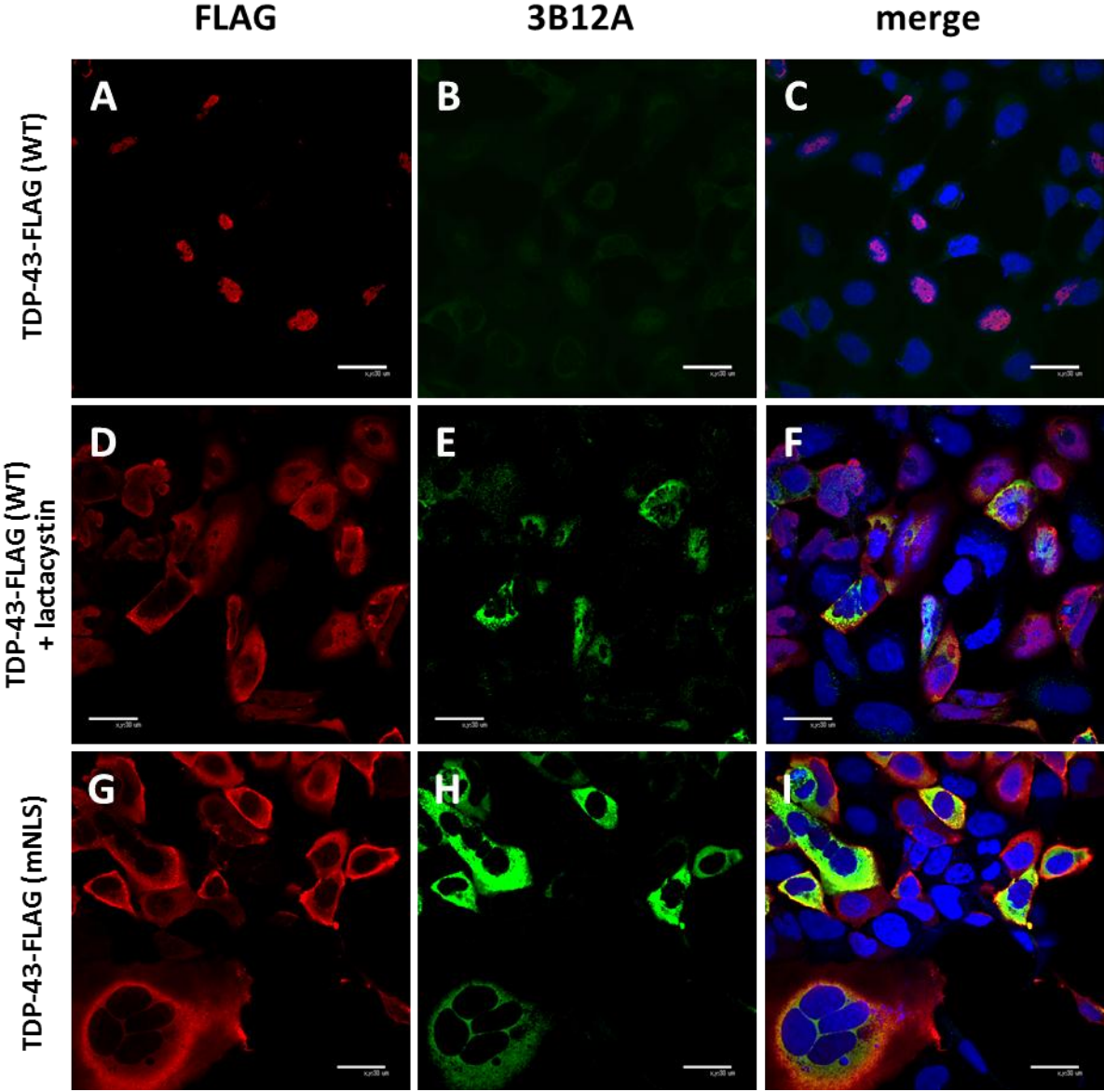

Supplement: Figure S5 — 3B12A mAb effectively stains cytosolic TDP-43. Confocal immunofluorescent micrographs of HEK293A cells expressing TDP-43-FLAG of wild-type (A, D) and defective NLS (mNLS, H). In D–F, HEK293A cells were treated with 20 mM lactacystin to induce nuclear exclusion of WT TDP-43. Cells were doubly stained with antibodies against FLAG (red) and 3B12A (green). Scale bar indicates 10 µm. (PDF) [file pone.0052776.s005.pdf]

Figure S6

A

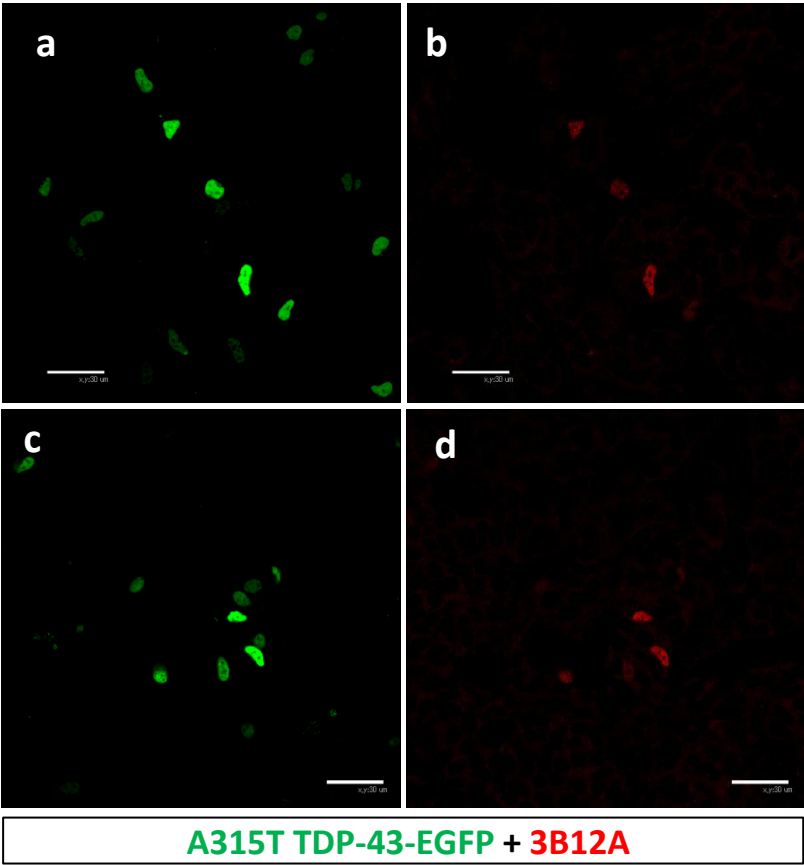

B

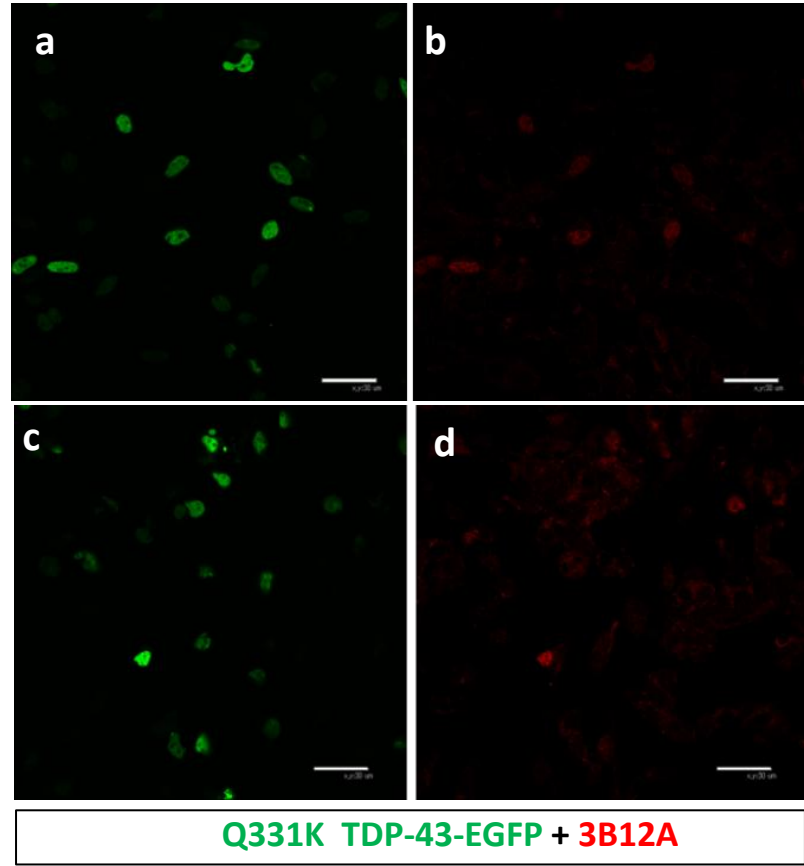

Supplement: Figure S6 — Familial ALS-linked TDP-43 is marginally recognized by 3B12A. Confocal immunofluorescent micrographs of SHSY-5Y cells expressing mutant TDP-43-EGFP (green, A, A315T; B, Q331K) which were stained by 3B12A mAb(red). Scale bar indicates 30 µm. (PDF) [file pone.0052776.s006.pdf]

**Figure S7**

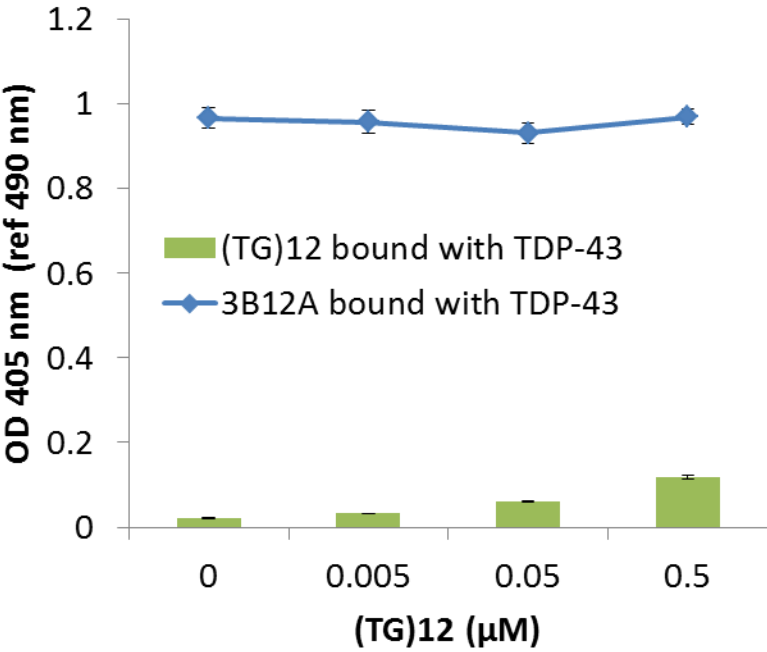

Supplement: Figure S7 — 3B12A mAb interacts with TDP-43 regardless of DNA interaction. A sandwich ELISA showing that 3B12A recognizes recombinant TDP-43 protein regardless of its interaction with biotin-labeled (TG)12 repeat oligonucleotides. Rabbit polyclonal anti-TDP-43 antibody was coated onto ELISA plate to capture recombinant TDP-43 protein. 3B12A was used for detection antibody after 1 hr incubation with biotin-labeled (TG)12 oligonucleotide (blue). The (TG)12, which interacted with captured TDP-43 was also quantified by obtaining peroxidase activity of streptavidin-conjugated horseradish peroxidase without 3B12A application (green). (PDF) [file pone.0052776.s007.pdf]

**Figure S8**

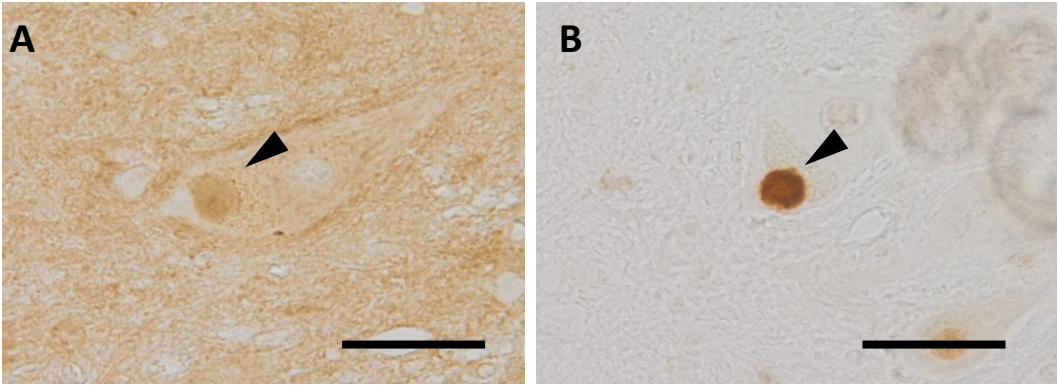

Supplement: Figure S8 — 3B12A stains TDP-43–positive round cytosolic inclusions. Serial sections from a different ALS patient from those in Fig. 6 were stained with 3B12A mAb (A) and anti-TDP-43 antibody (B). The round inclusion in the cytosol of motor neurons is immunoreactive to anti-TDP-43 and 3B12A antibodies. Scale bar indicates 50 µm. (PDF) [file pone.0052776.s008.pdf]

Figure S9

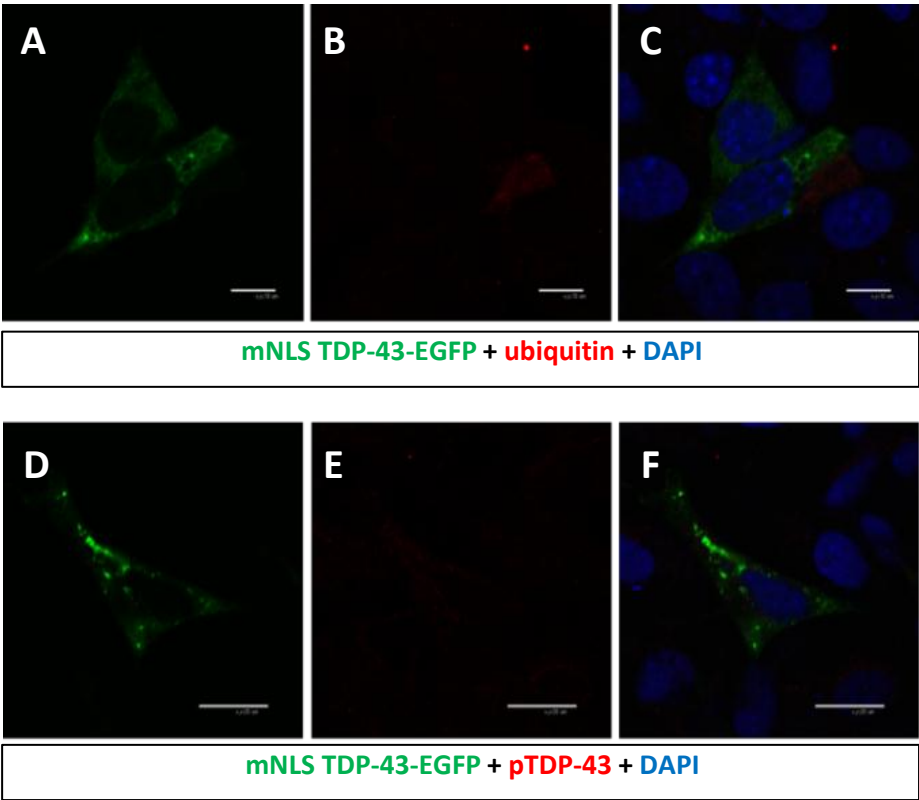

Supplement: Figure S9 — Non-aggregated forms of cytosolic TDP-43 are not ubiquitinated or phosphorylated. Confocal micrographs used for immunofluorescence analysis of SHSY-5Y cells that were transiently transfected with TDP-43-EGFP or the defective nuclear localizing signal form of TDP-43-EGFP (mNLS). After fixation with 4% paraformaldehyde, cells were stained with anti-ubiquitin (A–C) or anti-phosphorylated TDP-43 at S409/S410 (D–F) antibody. Note that the mNLS mutant form of TDP-43 is not labeled by these antibodies. Scale bar indicates 10 µm. (PDF) [file pone.0052776.s009.pdf]
