# Supplementary material for: Conserved Acidic Amino Acid Residues in a Second RNA Recognition Motif Regulate Assembly and Function of TDP-43
Source: PLoS One. 2012 Dec 26;7(12):e52776. doi: 10.1371/journal.pone.0052776 (PMC3530536; doi:10.1371/journal.pone.0052776)
Supplement: Table S1 — Primer pairs used for plasmid construction. (PDF) [file pone.0052776.s010.pdf]

Supplementary table 1

Primer pairs		
1. RRM2 ligation into pGEX6p-1		
	sense	gggatccccggaattcagcagaaaagtgtttgtgg
	antisense	gtcgacccgggaattcttaattgtgcttaggttcggca
2. mutation of E246 and D247		
E246G	sense	cgcagtctctttgtggagggggcttgatcattaaaggaatc
	antisense	gattcctttaatgatcaagccccctccacaaagagactgcg
D247G	sense	cagtctctttgtggagagggcttgatcattaaaggaatca
	antisense	tgattcctttaatgatcaagccctctccacaaagagactg
E246G/D247G	sense	cgcagtctctttgtggagggggcttgatcattaaaggaatc
	antisense	gattcctttaatgatcaagccccctccacaaagagactgcg
E246Q/D247N	sense	gattgcgcagtctctttgtggacagaacttgatcattaaaggaat
	antisense	attcctttaatgatcaagttctgtccacaaagagactgcgcaatc
3. Deletion mutants of TDP-43		
del RRM1	sense	caagatgagcctttgagaa
	antisense	tttctggactgctcttttc
del RRM2	sense	agcaatagacagttagaaa
	antisense	tctcaaaggctcatcttgg
