# Supplementary material for: Conserved Acidic Amino Acid Residues in a Second RNA Recognition Motif Regulate Assembly and Function of TDP-43
Source: PLoS One. 2012 Dec 26;7(12):e52776. doi: 10.1371/journal.pone.0052776 (PMC3530536; doi:10.1371/journal.pone.0052776)
Supplement: File S1 — Supplementary Materials and Methods. (PDF) [file pone.0052776.s011.pdf]

File S1 SUPPLEMENTARY INFORMATION FOR MATETIALS AND METHODS

Perfluoro-octanoic acid (PFO)-PAGE

PFO is a non-dissociative detergent that preserves protein-protein interactions of both cytosolic and membranous proteins (1). Protein samples in PBS were mixed with the same volume of PFO sampling buffer (100 mM Tris base, 2% NaPFO, 20% glycerol, 0.005% Bromophenol Blue, pH 8.0), and then incubated for 1 h at 22 oC. For the electrophoresis, a running buffer comprising 0.5% NaPFO, 25 mM Tris-HCl, and 192 mM glycine at pH 8.5 was pre-chilled, and protein samples were then separated using a 15% gradient Tris-Glycine polyacrylamide gel (Invitrogen) at 140 V with constant cooling on ice.

Enzyme-linked immunosorbent assay (ELISA)

Antibody titration was performed by ELISA using a previously reported protocol (26). In brief, target proteins in coating buffer (Roche) were coated onto an ELISA plate (Nunc, Rochester, NY) at a concentration of 1 g/ml. Coated proteins were then incubated with primary antibodies and subsequently with peroxidase-conjugated secondary antibody (Jackson Immunoresearch). Finally, the reaction buffer containing 2,2'-azino-bis[3-ethylbenzothiazoline-6-sulfonate] (ABTS, Roche) was applied, and the antibody titer was measured by a spectrometer at 405 nm with a reference of 490 nm.
A sandwich ELISA was employed to investigate whether the DNA interaction affects the immunoreactivity of 3B12A mAb with TDP-43. First, rabbit polyclonal anti-TDP-43 (raised against 1–261 aa; Proteintech) antibody (2 μg/ml) were coated as a capture antibody. After blocking, recombinant TDP-43-FLAG proteins WT were applied for 1 h at 22 ^{o}C. After three times washes, captured proteins were incubated with biotin-conjugated thymine-guanine (TG)12 repeated oligonucleotides for 1h at 37 ^{o}C. Finally, proteins were reacted with 3B12A mAb and then peroxidase-conjugated goat anti-mouse IgG as mentioned above. The efficiency of (TG)12 interaction with recombinant TDP-43 proteins on the ELISA plate was estimated by the reaction with horseradish peroxidase-conjugated streptavidin (Invitrogen) followed by incubation with ABTS in the absence of 3B12A mAb.

References

1. Ramjeesingh, M., Huan, L. J., Garami, E., and Bear, C. E. (1999) *Biochem. J.* **342 (Pt 1)**, 119-123
